# Extensive skin necrosis after periprosthetic knee infection: a case that highlights the possibility of complications induced by low-molecular-weight heparin

**DOI:** 10.5194/jbji-6-235-2021

**Published:** 2021-06-28

**Authors:** Hélder Fonte, André Carvalho, João Rosa, Cláudia Pereira, Alexandre Pereira, Ricardo Sousa, further members of the Porto Bone and Joint Infection Unit

**Affiliations:** 1 Department of Orthopaedics, Centro Hospitalar Universitário do Porto, Porto, Portugal; 2 Department of Internal Medicine, Centro Hospitalar Universitário do Porto, Porto, Portugal; 3 GRIP (Porto Bone and Joint Infection Unit), Centro Hospitalar Universitário do Porto, Porto, Portugal; ➕ A full list of authors appears at the end of the paper

## Abstract

We describe a case of a patient with atrial fibrillation, anticoagulated with dabigatran, that developed severe knee skin necrosis in the setting of an acute periprosthetic knee infection, after initiating low-molecular-weight heparin. A wide range of etiology hypotheses was discussed within a multidisciplinary team. The complex approach consisted of treating the underlying infection, multiple types of soft-tissue management, and stopping enoxaparin.

## 1 Introduction

Severe wound complications following total knee arthroplasty (TKA), though uncommon, are of major importance. Clinical presentation ranges from wound problems and superficial infections to full-thickness skin necrosis (Galat, 2009). One of the possible causes of skin necrosis is the administration of low-molecular-weight heparin. This complication, occurring at a distance from the injection site, has been increasingly reported and more frequently observed with subcutaneous therapy, though it is an extremely rare event. Female gender, high BMI (> 25 kg m${}^{-2}$), and long duration of heparin therapy have been identified to be the risk factors; however, little is known about the true incidence (Schindewolf et al., 2009). These mechanisms are considered: immunologically mediated either via thrombosis resulting from heparin-induced immune aggregation of platelets (heparin-induced thrombocytopenia syndrome, HIT) or a formation of antigen–antibody complexes in cutaneous blood vessels (type III hypersensitivity syndrome) (Handschin et al., 2005). Moreover, to the best of our knowledge, there is only one case reported in the English literature about tissue necrosis occurring after knee arthroplasty (Karmegam and Raut, 2011). The authors describe a case of acute knee periprosthetic joint infection (PJI) complicated with severe skin necrosis in an anticoagulated patient that stopped dabigatran before the surgery and started enoxaparin on the post-operative period. PJI treatment required a two-stage approach with implant removal, medial gastrocnemius muscle flap, negative pressure dressing, hyperbaric oxygen chamber, and split-thickness skin graft. After laborious soft-tissue management, the patient ultimately underwent a successful second-stage procedure, being able to walk autonomously and presenting a good knee range of motion, with complete wound healing.

**Figure 1:**
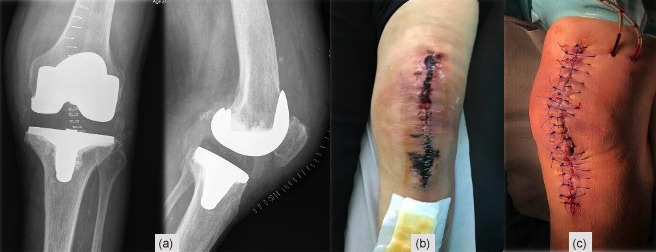
**(a)** Post-operative TKA X-ray, **(b)** skin necrosis on the 18th post-operative day, and **(c)** surgical wound immediately after debridement and antibiotics with implant retention (DAIR).

**Figure 2:**
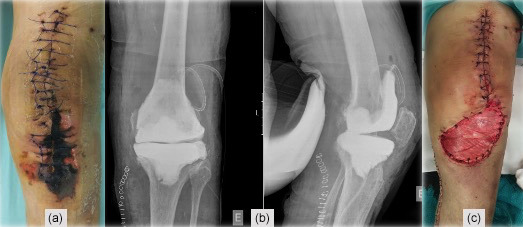
**(a)** Skin necrosis on the fifth day after DAIR, **(b)** X-ray after the implant removal and application of an antibiotic-loaded cement spacer, and **(c)** medial gastrocnemius muscle flap for wound coverage.

## 2 Report of the case

A 78-year-old woman, with a history of atrial fibrillation and hypertension, underwent left TKA for primary osteoarthritis (Fig. 1a). Chronic medication included dabigatran, olmesartan–hydrochlorothiazide, and amlodipine. There was no history of renal failure, diabetes mellitus, autoimmune disease, peripheral arterial or venous disease, or other relevant comorbidities (CHA2DS2-VASc = 4 points). Dabigatran was stopped 3 d before surgery. Throughout the procedure, a tourniquet was used (45 min with a pressure of 300 mm Hg), and a suction drain was in place for 24 h. During the post-operative period, therapeutic enoxaparin (60 mg BID) was started. The patient was discharged on day five with a clean wound and recommendation to switch back to oral dabigatran. On the 18th post-operative day, a significant wound skin necrosis was found with slight concurrent wound leakage (Fig. 1b), and despite C-reactive protein (CRP) levels of 15.8 mg L${}^{-1}$, there was a high suspicion of an underlying acute periprosthetic joint infection. The patient was switched back to enoxaparin and was scheduled for surgery within 2 d (Fig. 1c). After surgical debridement with implant retention (DAIR), broad-spectrum IV antibiotics were started (vancomycin and piperacillin–tazobactam). Despite these efforts, skin necrosis progressed on the distal part of the wound (Fig. 2a). Microbiological samples confirmed infection with *Enterococcus faecalis* and *Staphylococcus haemolyticus* in multiple samples. On the fifth day after DAIR, skin necrosis at the distal part of the wound progressed (Fig. 2a), and a decision was made to remove the prosthesis and implant an antibiotic-loaded handmade mobile spacer (with 4 g vancomycin and 2 g meropenem per bag of bone cement) and perform a medial gastrocnemius muscle flap for wound coverage (Fig. 2b–c). Microbiological samples, including implant sonication, isolated the same microorganisms, but given the unfavourable clinical course, broad-spectrum antibiotics were maintained for 6 weeks.

**Figure 3:**
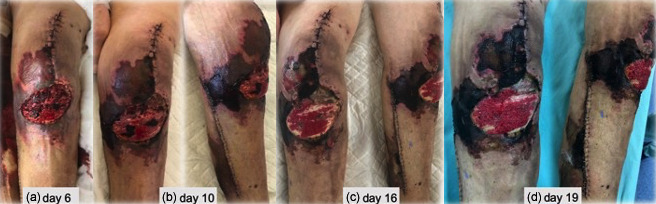
**(a–d)** Skin necrosis progression after TKA revision, extending to the lower leg around the incision made to harvest the muscle flap.

**Figure 4:**
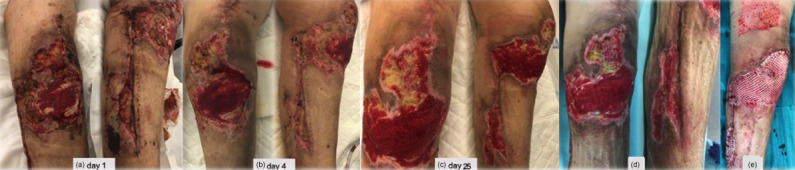
**(a)** A period of 3 weeks after revision surgery, new debridement of all necrotic areas, interruption of enoxaparin, negative pressure dressings, and hyperbaric oxygen chamber treatment were performed. **(b–c)** Progressive healing of the wound. **(d)** New debridement. **(e)** Split-thickness skin graft of the thigh for wound coverage.

During the next weeks, an extensive superficial skin necrosis developed around the knee, extending to the lower leg around the incision made to harvest the muscle flap (Fig. 3). An arteriography was performed, and occlusive arterial disease was definitively ruled out. Skin biopsy revealed microvascular thrombotic phenomena and ischemia without vasculitis. Complete blood count (CBC) revealed persistently normal platelet values and slight post-operative anaemia. Erythrocyte sedimentation rate (ESR) and C-reactive protein (CRP) levels were elevated but ran a favourable downward trend. All coagulation parameters were normal, and autoimmune disease assessment was negative. A haematology consult was also requested, thinking of low-molecular-weight heparin (LMWH)-induced skin necrosis, but given the lack of thrombocytopenia and atypical clinical presentation, a decision was made not to pursue more specific testing regardless of our suspicion.

After 3 weeks, surgical debridement of the necrotic tissues was undertaken. With the lack of an alternative diagnosis, a decision was made to discontinue enoxaparin and reinstate dabigatran. A negative-pressure dressing was applied, and complementary treatment on a hyperbaric oxygen chamber was also initiated. Necrosis stopped progressing (Fig. 4a–d), and eventually a split-thickness skin graft of the thigh for wound coverage was performed (Fig. 4e).

**Figure 5:**
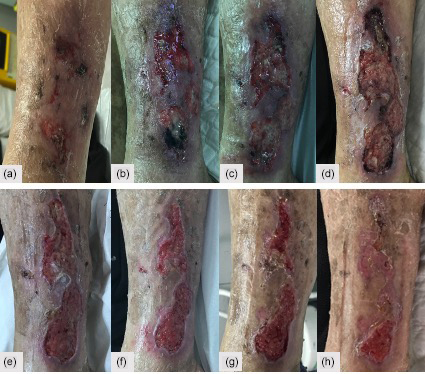
**(a–d)** Lateral leg wound evolution and **(e–h)** response after institution of a 2-week course of oral amoxicillin–clavulanate.

During the outpatient follow-up, soft tissues showed slow but progressive improvements and eventually healed completely. A few weeks after hospital discharge, an ulcer on the lateral aspect of the leg (unrelated to previous incisions) developed and progressed (Fig. 5a–d). A biopsy was again performed that revealed unspecific findings, suggesting an infected venous leg ulcer, and a 2-week course of oral amoxicillin–clavulanate was instituted with excellent clinical response (Fig. 5e–h). A new immunological assessment showed positive ANA with a low value of anti-dsDNA, without complement consumption and positive IgG anti-beta-2-glycoprotein I (anti-cardiolipin and lupus anticoagulant remained negative), but after 12 weeks they were back to normal.

**Figure 6:**
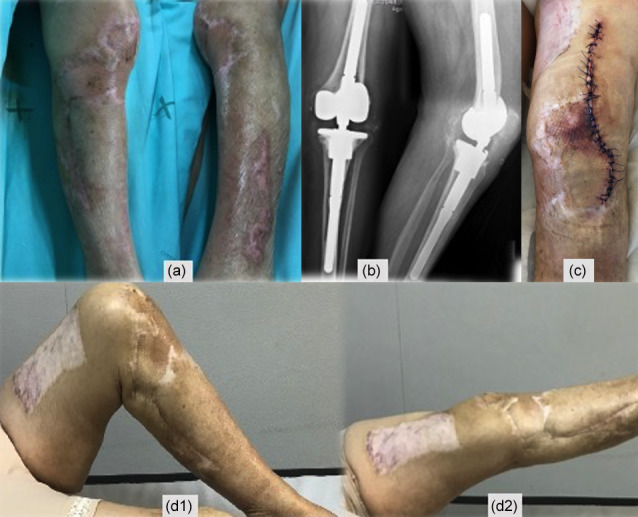
**(a)** Skin before the second-stage surgery, **(b)** post-operative X-ray, **(c)** skin fully healed, **(d1–d2)** left knee range of motion in the last evaluation, 0–115${}^{\circ }$.

Second-stage surgery was consecutively postponed, but when satisfactory soft tissues were present (Fig. 6a) and after an adequate discussion with the patient, a decision to go ahead with the second stage was taken 1 year after the original surgery. Dabigatran was discontinued for 3 d before, without enoxaparin bridging, and immediately resumed after surgery. Intraoperatively, multiple deep tissue samples were collected and showed no bacterial growth. A period of 2 years after the second stage, the patient is infection-free, with a painless, well-functioning knee arthroplasty, and no systemic disorder was diagnosed (Fig. 6).

## 3 Discussion

Extensive skin necrosis can originate in a wide range of etiologies, detailed in the following subsections.

### 3.1 Drug-induced skin necrosis

Skin necrosis in response to anticoagulant treatment is a rare adverse reaction and is even more uncommon in patients treated with heparin compared to oral anticoagulants (0.01 % of patients) (Estébanez et al., 2019). There are some pathophysiological mechanisms that explain LMWH-induced skin necrosis: first, it was found to be associated with an established heparin-induced thrombocytopenia (HIT) syndrome. Here, an antibody–platelet–heparin complex leads to an activation of the coagulation cascade that results in microthrombosis of dermal vessels and skin necrosis. Second, vasculitis of dermal vessels induced by a type III hypersensitivity reaction to the LMWH (Arthus phenomenon, with deposit of immunocomplexes on the endothelial structure) has been proposed as an alternative pathomechanism (Handschin et al., 2005). It usually presents close to the injection site, although it has been described at distance on rarer occasions (Balestra et al., 1994; Estébanez et al., 2019). To the best of our knowledge, there is only one reported case in the English literature about tissue necrosis occurring after knee arthroplasty (Karmegam and Raut, 2011). Despite the atypical location of the necrotic lesions and the lack of classical thrombocytopenia and heparin–platelet factor 4 antibodies, we believe this is a case of LMWH-induced skin necrosis for the following reasons:

1. Skin necrosis stopped progressing after we decided to discontinue enoxaparin and switch back to dabigatran.
2. Although the mechanism is not as clear, heparin-induced necrosis can occur in the absence of thrombocytopenia and responsible antibodies.
3. This is indeed an exclusion diagnosis, but we believe all other possible diagnoses were thoroughly excluded.

### 3.2 Infection

We believe it is imperative to address periprosthetic joint infection (PJI) early and aggressively to obtain good results (Barros et al., 2019). This principle was also observed in this case, and polymicrobial PJI was confirmed. Necrotizing fasciitis (NF) may also be caused by polymicrobial infections (Steer et al., 2012) and was considered despite a non-compatible clinical presentation. As such, despite the favourable course of blood inflammatory markers, we worried that some “occult” microorganism would be responsible for the ongoing skin necrosis and decided to keep broad-spectrum antibiotics for the entire 6-week period. The reasons that we believe skin necrosis was not caused by the underlying infection (and no reason to prolong antibiotic therapy was present) are as follows:

1. PJI was adequately addressed from the start. Debridement and antibiotics with implant retention were performed timely, and even if a persistent infection could be considered as a contributing cause in the early stages, it would certainly not be responsible for what happened after prosthesis removal and spacer implantation.
2. NF initial clinical presentation resembles cellulitis that rapidly progresses within 24 to 72 h (Steer et al., 2012). This was not the case here, nor was there ever disproportionate pain and tenderness compared with physical findings (cardinal finding) (Dahl et al., 2002). Moreover, the biopsy did not reveal any suggestive features.
3. Although skin necrosis of the distal part of the wound after DAIR may have been caused by wound closure with inadequate skin tension, that would not explain the skin necrosis after the original procedure or after the first stage and muscle flap.

### 3.3 Arterial disease or embolic phenomena

Although skin necrosis is the final result of superficial microvasculature occlusion, we worried that some kind of major arterial disease could be responsible for the exuberant clinical presentation. Blood vessel obstruction due to embolic phenomena was also a possible cause. The reasons that we believe skin necrosis was not caused by underlying arterial disease or embolic phenomena are as follows:

1. The patient underwent a completely normal angiography.
2. Completely normal laboratory coagulation parameters and continuing anticoagulation medication exclude a primary hypercoagulable condition as the cause for skin necrosis.
3. Unlike other forms of necrosis, with embolic phenomena, the areas of involvement tend to be small, distal, and multiple.

### 3.4 Autoimmune diseases

Several autoimmune diseases may present cutaneous involvement with skin necrosis. They could be associated with a positive ANA test (like systemic lupus erythematosus – SLE – and scleroderma) or ANCA-positive and ANCA-negative vasculitis. Antiphospholipid syndrome (APS) is an acquired thrombophilia caused by autoantibodies against phospholipids, causing arterial and venous thrombosis. Diagnosis of APS involves the presence of thrombotic clinical events in addition to elevated autoantibodies on at least two occasions, 12 weeks apart (Frances, 2010). The reasons that we believe it is not an autoimmune disease are as follows:

1. ANA and dsDNA were positive in the second study only (and not during acute post-operative skin necrosis), without systemic symptoms or laboratory findings to support the diagnosis of SLE. In addition, immunosuppression was not used in the acute event, and 2 years later, no other manifestations occurred.
2. Vasculitis is completely absent in all biopsies taken.
3. The absence of previous thrombotic events, other suggestive clinical features, and the presence of positive anti-beta2-glycoprotein in only one study do not support the diagnosis of APS.

### 3.5 Pyoderma gangrenosum

Pyoderma gangrenosum (PG) is an ulcerating neutrophilic dermatosis that can occur in areas of trauma or following surgical procedures. While the onset of PG is sudden, it tends to remain a chronic ailment. Diagnosis is often by exclusion, and although lesions appear, infected cultures are not useful (Ahronowitz et al., 2012). The reasons that we believe it is not PG are as follows:

1. After surgical debridement of the skin necrosis, there was no further progression of the ulcer. If PG was the culprit, one could expect post-surgical worsening, known as the pathergy phenomenon (Duarte et al., 2009), the reason that surgical debridement is usually contraindicated.
2. The spontaneous improvement despite the lack of corticosteroid/immunosuppressive therapy also speaks against this diagnosis, as does the good clinical outcome after the second-stage revision surgery.

This report illustrates a challenging case of extensive necrosis complicating a PJI. Difficulties around diagnosis and treatment were numerous, even within a well-trained multidisciplinary team. Despite a successful outcome, the exact aetiology of the necrosis remains unproven, but enoxaparin-induced skin necrosis emerges as a diagnostic of exclusion.

## Data Availability

The data used to support the findings of this study are included in the article.

## Team list

GRIP (Porto Bone and Joint Infection Unit) consists of the following members: Cláudia Pereira, Miguel Abreu, Daniel Soares, Ernestina Reis, Ana Cláudia Santos, and Ricardo Sousa.
